# Who Experiences Burden? A Typology of Dementia Care Dyads During Post-Hospital Transition

**DOI:** 10.1093/geroni/igaf122.1958

**Published:** 2025-12-31

**Authors:** Marie Boltz

**Affiliations:** Pennsylvania State University, University Park, Pennsylvania, United States

## Abstract

Dyads of persons living with dementia and their care partners represent diverse populations with unique characteristics and needs. The everyday stress faced by care partners is compounded by new burdens after hospitalization of the person with dementia. This study aimed to develop a typology of dyads based on characteristics of the care partner, as well as their care receivers, to gain a better understanding of the association of dyad typology with care partner burden during transition from the hospital. A latent class analysis was used to detect different dyad types based on intrinsic characteristics of 451 dyads of care partners and their care receivers, persons living with dementia. The care partners’ average age was 61.8 (SD = 14.1); 291 (63%) were white; 154 (33%) were Black and the majority were daughters (n = 172, 37.3%) or spouses (n = 132, 28.6%), who lived with the care receiver (60%) and perceived their health as good or better. Care receivers’ average age was 81.6 (8.4); they demonstrated moderate cognitive impairment (MoCA mean=11.6, SD = 7.0) and 4 (SD = 2.8) co-morbidities on average. A 5-class model was identified. The classes could be differentiated based on care partner- care receiver key characteristics (gender, age, kinship, living situation, health). There were significant differences with regard to burden. The verification of different types of dyads strengthens the need to develop tailored dyad-centered interventions to support the care partner of the person with dementia post-hospitalization.

